# Clinicopathological diagnostic concordance among autopsied adults living with HIV in the Brazilian Amazon: A retrospective cross-sectional study

**DOI:** 10.1371/journal.pone.0361097

**Published:** 2026-10-26

**Authors:** Yasmin Ferreira Martins, Taynná Vernalha Rocha Almeida, Monique Freire dos Reis, Vanessa Vieira Pinheiro Corrêa, Eveny Perlize Melo Marinho, Rebecca Augusta de Araújo Pinto, Michele de Souza Bastos, Luiz Carlos de Lima Ferreira

**Affiliations:** 1 Fundação de Medicina Tropical Doutor Heitor Vieira Dourado, Manaus, Amazonas, Brazil; 2 Universidade Federal do Amazonas, Programa de Pós-Graduação em Imunologia Básica e Aplicada, Manaus, Amazonas, Brazil; 3 Universidade Federal do Amazonas, Programa de Pós-Graduação em Ciências da Saúde, Manaus, Amazonas, Brazil,; 4 Universidade do Estado do Amazonas, Programa de Pós-Graduação em Medicina Tropical, Manaus, Amazonas, Brazil; 5 Departamento de Ensino e Pesquisa, Fundação Centro de Controle de Oncologia do Estado do Amazonas – FCECON, Manaus, Amazonas, Brazil; 6 Departamento de Patologia e Medicina Legal, Hospital Universitário Getúlio Vargas, Universidade Federal do Amazonas, Manaus, Amazonas, Brazil; 7 Universidade do Estado do Amazonas, Manaus, Amazonas, Brazil; University of California San Diego, UNITED STATES OF AMERICA

## Abstract

With the emergence of antiretroviral therapy (ART), changes have occurred over time in the causes of death and pathological findings among people living with HIV, including an increasing contribution of serious non-AIDS-related diseases. Assessing concordance between ante-mortem clinical and post-mortem diagnoses remains an important approach for evaluating diagnostic performance and monitoring changes in causes of death in this population. This study aimed to evaluate the concordance between ante-mortem clinical diagnoses of the underlying cause of death and autopsy diagnoses among adults living with HIV who underwent autopsy at the Fundação de Medicina Tropical Doutor Heitor Vieira Dourado (FMT-HVD), Manaus, Brazil, between 2014 and 2019. This cross-sectional retrospective study included 101 autopsies. Class I discordances were identified in 48 of 101 cases (47.6%), predominantly involving infectious diseases (37/48; 77.1%), followed by undiagnosed neoplasms (8/48; 16.7%) and other conditions (3/48; 6.2%). Overall concordance for the underlying cause of death was 36.7%, and most deaths were attributable to AIDS-defining diseases. The absence of ART was associated with a higher frequency of class I discordances. The high frequency of major missed diagnoses may also reflect advanced disease at presentation, overlapping clinical manifestations of opportunistic infections, and limitations in the availability of specific diagnostic methods during the study period. Overall clinicopathological discordance was 63.3%, underscoring the continued value of autopsy for identifying missed diagnoses and highlighting the importance of timely clinical assessment and improved access to sensitive and specific diagnostic methods for adults living with HIV.

## Introduction

Although antiretroviral therapy (ART) has substantially reduced HIV-related mortality, approximately 630,000 people died from AIDS-related diseases worldwide in 2023 [1]. The expansion of ART has also changed patterns of morbidity and mortality among people living with HIV, with a decline in several opportunistic infections and an increasing contribution of serious non-AIDS-related conditions [2–4].

Autopsy remains an important reference for determining causes of death and identifying clinically unsuspected conditions. Despite advances in diagnostic methods and declining autopsy rates, discrepancies between ante-mortem and post-mortem diagnoses remain frequent, including among people living with HIV [5–8]. In a recent autopsy study conducted at a tertiary hospital in the United States using the Goldman classification criteria [9,10], class I discordances occurred in 9.9% of cases, with untreated infections accounting for most major missed diagnoses. These findings demonstrate the continued value of autopsy for identifying clinically relevant diagnostic discrepancies despite advances in clinical investigation.

Previous autopsy-based research conducted at FMT-HVD evaluated causes of death and clinicopathological concordance among individuals with AIDS who died between 1996 and 2003, reporting an overall concordance of 51.9% between ante-mortem and post-mortem diagnoses [11]. However, patterns of HIV-related morbidity and mortality, access to ART, and diagnostic resources have changed substantially since that period. An updated assessment is therefore warranted to determine whether major diagnostic discrepancies persist in a more recent period of HIV care in the Brazilian Amazon. The present study extends the previous institutional analysis by applying the Goldman classification to assess the clinical relevance of diagnostic discrepancies and by examining their relationship with ART status, CD4 cell count, viral load, and length of hospitalization.

This study aimed to evaluate concordance between ante-mortem clinical and post-mortem diagnoses of causes of death among adults living with HIV who underwent autopsy at a reference center for tropical diseases in the Brazilian Amazon. Secondary objectives were to classify diagnostic discrepancies according to the Goldman criteria; assess concordance for the underlying and immediate causes of death; and explore the relationships of diagnostic discrepancies with ART status, CD4 cell count, viral load, and length of hospitalization.

## Materials and methods

This is a cross-sectional and retrospective study (S1 STROBE Statement) carried out at the Fundação de Medicina Tropical Doutor Heitor Vieira Dourado (FMT-HVD), a national and international reference center for the treatment of tropical diseases, located in the city of Manaus, state of Amazonas.

The source population comprised adults living with HIV who died at FMT-HVD between January 2014 and December 2019. Mortality records for the study period were obtained from the Mortality Information System (SIM), provided by the Escola de Saúde Pública de Manaus (ESAP), under the Municipal Health Secretariat of Manaus (SEMSA). During the study period, autopsy was routinely offered to the families of deceased patients at FMT-HVD, without specific institutional criteria for selecting cases for autopsy. Thus, there was no institutional policy preferentially selecting cases for autopsy on the basis of diagnostic uncertainty or other predefined clinical circumstances. The study sample was restricted to adults living with HIV who underwent autopsy at FMT-HVD during the study period. Individuals aged 18 years or older were eligible for inclusion. Cases were excluded if medical records or relevant clinical information could not be retrieved, if the individual underwent autopsy at FMT-HVD but had not received care at the institution, if the autopsy report was unavailable, or if the autopsy findings were inconclusive.

### Data collection

Clinical, sociodemographic, and cause-of-death diagnostic data were collected exclusively from pre-existing records, including electronic medical charts from the FMT-HVD internal system and information from the Mortality Information System (SIM) provided by the Escola de Saúde Pública de Manaus (ESAP), under the Municipal Health Secretariat of Manaus (SEMSA). Clinical diagnoses prior to death were made by the attending physicians during hospitalization, based on clinical evaluation, patient history, and available laboratory and imaging tests. The underlying and immediate causes of death were determined from the medical records and death certificates issued by the attending physicians. Autopsy data were retrieved from archived reports available at the Pathology Department of FMT-HVD. All data accessed were fully anonymized prior to analysis, and no information that could identify individual participants was collected at any stage of the study.

As this was a retrospective study using only anonymized medical and autopsy records, the Research Ethics Committee of FMT-HVD approved the study and waived the requirement for obtaining informed consent. Data extraction and organization were carried out between 16 November 2021 and 15 November 2022.

### Data analysis

The analysis of discordances regarding the underlying cause of death and the pathologies related and unrelated to death were carried out using the Goldman classification criteria [10] as used by ORDI et al. [12]. Cases were independently reviewed by two evaluators, including a senior pathologist, and categorized as major discrepancies (directly related to death: class I or II) or minor discrepancies (not directly related to death: class III or IV). In cases of disagreement between the two evaluators or uncertainty regarding the appropriate classification, a third pathologist was consulted, and the final classification was determined by the senior pathologist after consideration of the additional assessment.

Class I comprised major missed diagnoses directly related to death whose detection before death could have led to appropriate treatment capable of prolonging survival or curing the condition. For example, when pulmonary tuberculosis was considered the cause of death ante-mortem but autopsy revealed pulmonary histoplasmosis, the case was classified as class I if recognition of histoplasmosis during life could have resulted in specific treatment with the potential to alter the outcome. Class II included major missed diagnoses directly related to death whose detection would probably not have altered survival or clinical management. Class III comprised minor missed diagnoses unrelated to the primary cause of death but whose treatment could have influenced prognosis, whereas class IV included conditions of epidemiological or genetic importance without direct impact on patient outcome. Cases with no clinicopathological discrepancies were classified as class V, with the autopsy diagnosis considered the reference standard. When findings within a case could be assigned to more than one Goldman category, the highest-ranking class (the lowest numerical class) was assigned.

Overall clinicopathological concordance was defined as the proportion of cases classified as Goldman class V, whereas the overall discordance rate was defined as the proportion of cases classified as Goldman classes I–IV. For disease-specific agreement analyses, ante-mortem clinical and post-mortem diagnoses were paired at the individual level for all 101 cases and coded as present or absent for each condition. Clinically equivalent diagnostic terms were grouped into the same disease category for agreement analyses. Cohen’s kappa coefficient was used to assess agreement beyond chance. Kappa coefficients were reported for conditions in which both diagnostic sources showed variability in classification; for conditions with no positive diagnoses in one of the two sources, kappa coefficients were not reported because the absence of variability in one classification limits their interpretability.

Associations between Goldman classification and patient characteristics were assessed using the Kruskal–Wallis test for continuous variables (age, CD4 cell count, and viral load) and Fisher’s exact test for categorical variables (sex, comorbidities, and ART status). CD4 cell count and viral load were also compared according to the presence or absence of an AIDS-defining disease as the underlying cause of death using the Kruskal–Wallis test. When the Kruskal–Wallis test indicated a statistically significant difference, pairwise comparisons were performed using Dunn’s post hoc test with Holm correction for multiple comparisons. For significant Fisher’s exact tests, adjusted standardized residuals were examined to identify cells contributing to the association. The relationship between length of hospital stay and Goldman classification was assessed using Spearman’s rank correlation coefficient. All statistical analyses were performed using SPSS Statistics, version 26.

### Ethical considerations

This study was approved by the Human Research Ethics Committee of FMT-HVD accordingly CAAE: 68956117.2.0000.0005, No. 5.054.881.

## Results

According to mortality records provided by the Municipal Health Secretariat of Manaus (SEMSA), 1,318 deaths among adults living with HIV were recorded at FMT-HVD between 2014 and 2019. During the same period, 130 autopsies of adults living with HIV were identified in the institutional autopsy records. After application of the eligibility criteria, 101 cases were included in the final analysis.

Most individuals were from the state of Amazonas (86.1%), were male (70.3%), and had a median age of 34 years (range, 22–85). Fever, dyspnea, and cough were the most frequently recorded symptoms at admission. Regarding ART status, 39.7% reported regular use, 30.7% irregular use, and 26.7% were not receiving ART. The median length of hospitalization was 11 days (range, 1–180) (S1 and S2 Table).

Among individuals with available laboratory data, most had advanced immunosuppression, with a median CD4 cell count of 42 cells/µL (range, 2–501). The median detectable viral load was 110,608.5 copies/mL (range, 40–9,364,291), while 8.9% had an undetectable viral load (S3 Table).

Infectious diseases were the most frequent underlying causes of death (74.2%), particularly tuberculosis and bacterial pneumonia/bronchopneumonia (16 cases each), followed by neoplasms (13.9%) (Table 1). Overall, 62.4% of underlying causes of death were AIDS-defining conditions.

**Table 1:** Anatomopathological diagnoses of underlying cause of death of the 101 HIV patients autopsied at the FMT-HVD, between 2014 and 2019.

Category	N (%)
**Infectious diseases**	**75 (74,2)**
Tuberculosis	16
Disseminated	11
Pulmonary	5
Bacterial pneumonia/bronchopneumonia	16
Histoplasmosis	11
Disseminated	9
Pulmonary	2
Pneumocystosis	10
Cryptococcosis	8
Disseminated	6
Neurocryptococcosis	2
Cytomegalovirus pneumonia	4
Neurotoxoplasmosis	4
Viral encephalitis	2
Sepsis	2
Necrotizing pneumonia due to mucormycosis	1
*Strongyloides stercoralis* disseminated in lungs	1
**Neoplasms**	**14 (13,9)**
Kaposi’s sarcoma	7
Lymphomatoid granulomatosis	2
Diffuse large B-cell non-Hodgkin lymphoma	1
B lymphoblastic leucemia	1
Malignant breast neoplasm	1
Malignant neoplasm of the bile and extrahepatic ducts	1
Anaplastic thyroid carcinoma	1
**Other diseases**	**12 (11,9)**
Pulmonary hemorrhage + edema	6
Pulmonary hemorrhage	2
Extensive lung involvement due to fibrosis	2
Lung atelectasis	1
Dilated cardiomyopathy	1
**Total**	**101 (100)**

According to the Goldman classification, 48 of 101 cases (47.6%) had class I discordances, predominantly involving untreated infectious diseases (37/48; 77.1%), followed by undiagnosed neoplasms (8/48; 16.7%) and other conditions (3/48; 6.2%) (Tables 2 and 3). Class II, III, and IV discordances accounted for 7.9%, 5.9%, and 1.9% of cases, respectively, whereas 36.7% were classified as class V (no discordance). Detailed clinical and post-mortem findings for class I cases are provided in S2 Chart.

**Table 2:** Result of case classification according to Goldman criteria.

	Class	N (%)
Major discordances	I	48 (47,6)
	II	8 (7,9)
Minor discordances	III	6 (5,9)
	IV	2 (1,9)
No discordance	V	37 (36,7)
		
**Total**		**101 (100)**

**Table 3:** Anatomopathological findings in cases of major class I discordances.

Category	N (%)
**Infectious diseases**	**37 (77,1)**
Pneumocystosis	9
Histoplasmosis	8
Disseminated	6
Pulmonary	2
Bacterial pneumonia/bronchopneumonia	6
Cytomegalovirus pneumonia	4
Disseminated cryptococcosis	2
Viral encephalitis	2
Disseminated tuberculosis	2
Neurotoxoplasmosis	1
Necrotizing pneumonia due to mucormycosis	1
Pulmonary Tuberculosis	1
*Strongyloides stercoralis* disseminated in lungs	1
**Neoplasms**	**8 (16,7)**
Kaposi’s sarcoma	3
Lymphomatoid granulomatosis	2
Diffuse large B-cell non-Hodgkin lymphoma	1
B lymphoblastic leucemia	1
Anaplastic thyroid carcinoma	1
**Other diseases**	**3 (6,2)**
Pulmonary hemorrhage	2
Pulmonary hemorrhage + edema	1
**Total**	**48 (100)**

In cases where there were no discordances (class V), infectious pathologies predominated (67.6%), with tuberculosis being the most common, followed by bacterial pneumonia/bronchopneumonia and cryptococcosis (Table 4).

**Table 4:** Anatomopathological findings in cases without discordances (class V).

Category	N (%)
**Infectious diseases**	**25 (67,6)**
Tuberculosis	9
Disseminated	7
Pulmonary	2
Bacterial pneumonia/bronchopneumonia	6
Cryptococcosis	5
Disseminated	4
Neurocryptococcosis	1
Neurotoxoplasmosis	2
Sepsis	2
Disseminated histoplasmosis	1
**Other diseases**	**7 (18,9)**
Pulmonary hemorrhage + edema	5
Lung atelectasis	1
Dilated cardiomyopathy	1
**Neoplasms**	**5 (13,5)**
Kaposi’s sarcoma	3
Malignant breast neoplasm	1
Malignant neoplasm of the bile and extrahepatic ducts	1
**Total**	**37 (100)**

No statistically significant differences in Goldman classification were detected according to age (Kruskal–Wallis test, χ² [4]=1.54; p = 0.819), sex (Fisher’s exact test, χ² [4]=2.083; p = 0.747), or presence of comorbidities (Fisher’s exact test, χ² [4]=7.589; p = 0.069). In contrast, Goldman classification was associated with ART status (Fisher’s exact test, χ² [8]=15.723; p = 0.016). Adjusted residual analysis indicated that this association was primarily attributable to a higher frequency of class I discordances among individuals who were not receiving ART. No statistically significant differences across Goldman classes were detected for CD4 cell count (p = 0.440) or viral load (p = 0.225). Similarly, no statistically significant differences in CD4 cell count (p = 0.181) or viral load (p = 0.193) were detected according to the presence of an AIDS-defining disease as the underlying cause of death. Length of hospitalization showed a weak positive correlation with Goldman class (Spearman’s ρ = 0.201; p = 0.044). Given the small magnitude of the correlation, its clinical relevance should be interpreted cautiously.

Disease-specific agreement between ante-mortem clinical and post-mortem diagnoses was assessed using paired classifications for all 101 individuals. For each condition, the diagnosis was coded as present or absent in each source, thereby incorporating both concordant and discordant classifications into the analysis. Cohen’s kappa coefficients for the underlying and immediate causes of death are presented in Tables 5 and 6, respectively. Kappa coefficients were not reported for conditions with no positive diagnoses in one of the two sources, as the absence of variability in one classification limits the interpretability of the coefficient.

**Table 5:** Analysis of concordance between clinical and autopsy diagnoses regarding the declared underlying cause of death.

Disease	Diagnosis at autopsy	Clinical diagnosis	Concordance	Kappa
Tuberculosis	16	31	13	0,43
Bacterial pneumonia/bronchopneumonia	16	20	8	0,32
Neoplasms	14	7	6	0,52
Histoplasmosis	11	3	3	0,40
Pneumocystosis	10	3	1	0,11
Cryptococcosis	8	6	6	0,84
Pulmonary hemorrhage	8	5	5	0,75
Neurotoxoplasmosis	4	3	3	0,65
Sepsis	2	2	2	1,00

**Table 6:** Analysis of concordance between clinical and autopsy diagnoses regarding the declared immediate cause of death.

Disease	Diagnosis at autopsy	Clinical diagnosis	Concordance	Kappa
Acute Respiratory Failure	65	27	23	0,19
Sepsis	9	18	4	0,20
Intracranial hypertension	4	6	3	0,58
Toxoplasma meningoencephalitis	2	1	1	0,66
Pneumonia/bronchopneumonia	1	2	1	0,66

## Discussion

In this autopsy-based study of adults living with HIV at a reference center for tropical and infectious diseases in the Brazilian Amazon, substantial clinicopathological discordance was observed between ante-mortem and post-mortem diagnoses.

In our cohort, overall concordance between ante-mortem and post-mortem diagnoses of the underlying cause of death was 36.7%. Tuberculosis and bacterial pneumonia/bronchopneumonia were the most frequent causes of death (16 cases each), whereas pneumocystosis showed particularly low diagnostic agreement, with only one concordant case among ten identified at autopsy. The overall concordance was lower than the 51.9% reported by Souza *et al.* [11] in 129 autopsies previously conducted at the same institution. However, this comparison should be interpreted in light of the different periods of HIV care, as the previous cohort included deaths occurring between 1996 and 2003, when ART access and diagnostic resources differed substantially from those available during the period evaluated in the present study.

Opportunistic infections remained prominent in our cohort, in which most individuals had advanced immunosuppression and many were either not receiving ART or reported irregular use. Previous studies have shown that the spectrum of HIV-related mortality changes with effective ART, with reductions in several opportunistic infections as immune function improves [4]. Nevertheless, opportunistic infections may remain important causes of death in the ART era, particularly in settings where treatment is initiated late, adherence is suboptimal, or effective immune reconstitution has not been achieved [6,13]. These factors may partly explain the persistence of severe opportunistic diseases in our cohort; however, the retrospective nature of our data does not allow us to determine the individual contribution of late ART initiation, treatment adherence, or virological failure.

Class I discordances were identified in 47.6% of cases, while major discordances (classes I and II) accounted for 55.5%. Comparable rates have been reported in previous Brazilian autopsy studies. A retrospective study of 284 autopsied individuals with AIDS reported class I discrepancies in 53.8% of discordant cases and observed a reduction in class I errors over time, which the authors attributed in part to knowledge gained from previous autopsy findings and anatomoclinical discussions [14]. At FMT-HVD, Palhares *et al.* [15] reported major diagnostic discrepancies in 49% of 61 deaths evaluated by complete and minimally invasive autopsy, including 37 individuals living with HIV; 41% were classified as class I and 8% as class II. Although these findings indicate that high rates of major diagnostic discrepancies have also been documented in Brazilian autopsy cohorts, the 47.6% class I rate observed in our study remains substantial and warrants consideration of the clinical and diagnostic context in which these deaths occurred.

Tuberculosis and bacterial pneumonia/bronchopneumonia were the leading underlying causes of death in our cohort, consistent with reports from other settings with a high burden of HIV and infectious diseases, including India and sub-Saharan Africa [6,7,16,17]. Brazilian autopsy studies have similarly identified pulmonary infections as major causes of death, although their relative frequencies vary across cohorts [18]. Fungal infections also represented a substantial burden in our study, accounting for 29.7% of underlying causes of death, with histoplasmosis and pneumocystosis being the most frequent. This proportion was comparable to that reported in a previous autopsy study at FMT-HVD, in which fungal infections accounted for 27% of cases and showed a similar predominance of histoplasmosis and pneumocystosis [11], whereas other Brazilian cohorts have reported different distributions of fungal diseases [19].

Malignancies accounted for 13.9% of underlying causes of death, with Kaposi’s sarcoma being the most frequent, followed by lymphoproliferative disorders and other solid tumors. Both AIDS-defining cancers (ADCs), including Kaposi’s sarcoma and non-Hodgkin lymphoma, and non-AIDS-defining cancers (NADCs), such as breast cancer, cholangiocarcinoma, and anaplastic thyroid carcinoma, were identified in our cohort. This heterogeneous pattern is consistent with the changing spectrum of malignancies among people living with HIV in the ART era, characterized by a decline in several ADCs and an increasing relative burden of NADCs as survival improves [20–24]. Although infectious diseases remained the predominant causes of death in our cohort, the occurrence of both ADCs and NADCs highlights the importance of maintaining oncologic surveillance as part of comprehensive HIV care.

The high proportion of class I discordances observed in our study (47.6%) is likely multifactorial and should be interpreted in the context of the population and diagnostic resources available at FMT-HVD during the study period. Most class I discrepancies involved potentially treatable infectious diseases, particularly pneumocystosis, histoplasmosis, cytomegalovirus infection, and tuberculosis. Several factors may have contributed to these missed diagnoses, including advanced immunosuppression, overlapping clinical and radiological manifestations of opportunistic infections, and limited access to sensitive pathogen-specific diagnostic methods during the study period. In severely immunocompromised individuals, multiple opportunistic infections may also coexist, further complicating ante-mortem diagnosis.

Histoplasmosis and pneumocystosis illustrate these diagnostic challenges. Histoplasmosis may clinically and radiologically mimic tuberculosis, particularly in disseminated or pulmonary disease, which may favor empirical treatment for tuberculosis in endemic settings when confirmatory testing is unavailable or inconclusive [25–30]. In our cohort, histoplasmosis was frequently misclassified clinically as tuberculosis, pneumocystosis, or bacterial pneumonia. Importantly, direct Histoplasma antigen detection was not available at FMT-HVD during the study period and was implemented only in 2021. Pneumocystosis likewise presents with nonspecific respiratory manifestations and may coexist with other pulmonary infections, including cytomegalovirus infection, as observed in our cohort. Conventional microscopic diagnosis also has limited sensitivity and depends on appropriate respiratory sampling and experienced personnel, whereas more sensitive molecular methods were not routinely available during the study period [31–34]. These diagnostic limitations may therefore have contributed to the high frequency of major missed infectious diagnoses. These findings highlight the importance of considering multiple opportunistic infections early in the diagnostic evaluation of severely immunocompromised individuals, particularly when clinical or radiological findings are nonspecific or when there is no response to empirical therapy. Expanding access to rapid and sensitive pathogen-specific diagnostic methods, together with maintaining a broad differential diagnosis, may help reduce major missed diagnoses in this population.

The high class I discordance rate may also reflect characteristics of the population treated at FMT-HVD. The cohort included a substantial proportion of individuals with advanced immunosuppression, and absence of ART was associated with a higher frequency of class I discordance. Late presentation, lack of ART, irregular treatment, or insufficient immune recovery may increase both the burden and complexity of opportunistic diseases, making clinical diagnosis more challenging. However, these factors should be interpreted as plausible contributors rather than established causes of diagnostic discordance, as the retrospective design does not allow causal inference.

Comparisons with discordance rates reported by other institutions should be made cautiously. FMT-HVD is a tertiary reference center for tropical and infectious diseases in the Brazilian Amazon, and its patient population and diagnostic context may differ substantially from those of centers with broader access to advanced diagnostic technologies. In addition, the present analysis was restricted to individuals who underwent autopsy. Although autopsy was routinely offered to families without an institutional policy preferentially selecting cases on the basis of diagnostic uncertainty, only a subset of all deaths resulted in autopsy. Therefore, selection of the autopsied population may also have influenced the observed magnitude of discordance and limits direct comparison with other cohorts.

Among disease-specific analyses of the underlying cause of death, tuberculosis had the highest number of concordant diagnoses (13 cases), although four of these individuals were not classified as Goldman class V because additional clinically relevant diagnoses were missed. Cryptococcosis showed the strongest disease-specific agreement (κ = 0.84), likely reflecting the availability at FMT-HVD of established diagnostic methods, including cryptococcal antigen testing in serum, plasma, and cerebrospinal fluid. In contrast, despite having the highest absolute number of concordant diagnoses, tuberculosis showed only moderate agreement (κ = 0.43), partly because it was more frequently diagnosed ante-mortem than identified as the underlying cause at autopsy. The clinical overlap between tuberculosis and other opportunistic infections may have contributed to this pattern.

Length of hospital stay showed only a weak correlation with Goldman classification, suggesting that longer hospitalization did not necessarily translate into fewer diagnostic discrepancies. No statistically significant differences in Goldman classification were detected according to age, sex, comorbidities, CD4 cell count, or viral load, although these findings should be interpreted cautiously given the limited statistical power of subgroup analyses. In contrast, Goldman classification was significantly associated with ART status, with class I discordances occurring more frequently among individuals who were not receiving ART. This finding is clinically plausible, as absence of effective ART and advanced immunosuppression increase susceptibility to opportunistic diseases and may result in complex or concurrent clinical presentations. Consistent with this context, most individuals in our cohort had CD4 cell counts below 200 cells/µL, a level associated with increased susceptibility to several opportunistic infections and AIDS-related conditions [35].

The overall clinicopathological discordance rate in our study was 63.3%, higher than that previously reported at the same institution (48.1%) [11] and in cohorts from Italy (47%) [36], Uganda (45%) [6], and India (42%) [7], although lower than the 70% reported in England [8]. Direct comparisons across studies should be interpreted cautiously, as differences in study periods, patient populations, access to ART, diagnostic resources, autopsy practices, and definitions of discordance may influence the observed rates. In our setting, the predominance of advanced immunosuppression and opportunistic infections, together with the diagnostic challenges discussed above, may have contributed to the high discordance rate. However, because the present study included a selected autopsy cohort from a tertiary referral center for tropical and infectious diseases, the observed rate should not be interpreted as representative of all deaths among people living with HIV at FMT-HVD or directly generalized to other settings.

The main limitations of this study are inherent to its retrospective and single-center design. Clinical records and autopsy reports may contain incomplete or inconsistent information, and ART status was partly based on patient self-report, introducing the possibility of misclassification. In addition, as FMT-HVD is a reference center for tropical and infectious diseases in the Brazilian Amazon, our findings may not reflect the epidemiological profile of people living with HIV in other settings. The limited availability of advanced diagnostic methods during the study period, including molecular assays for fungal and viral infections, may also have contributed to underdiagnosis and, consequently, to the observed diagnostic discrepancies.

Autopsy selection bias should also be considered. Although autopsy was routinely offered to families without specific institutional criteria for case selection, only a subset of deaths among adults living with HIV resulted in autopsy. Therefore, individuals included in this study may not be fully representative of all adults living with HIV who died at the institution, and the observed discordance rates should not be generalized to all deaths in this population. Furthermore, the relatively small sample size limited the statistical power of some subgroup analyses to detect modest associations. Accordingly, non-significant findings, particularly those involving demographic, immunological, and virological characteristics, should not be interpreted as evidence of absence of an association and should be considered exploratory. In addition, the absence of a contemporaneous autopsy comparison group without HIV precluded assessment of whether the observed discordance rate was specific to adults living with HIV or reflected broader diagnostic challenges at the institution.

Despite these limitations, our findings show that major clinicopathological discordances remain frequent among autopsied adults living with HIV in this setting, predominantly involving potentially treatable opportunistic infections. The persistence of these discrepancies highlights the value of autopsy in identifying diagnostic gaps and informing improvements in clinical practice, particularly in settings with a high burden of infectious diseases. Recognition of recurrent patterns of missed diagnoses may also help guide diagnostic strategies and reinforce the continued role of autopsy in clinical education and quality improvement. Future prospective studies incorporating contemporary pathogen-specific diagnostic technologies are needed to determine whether the high clinicopathological discordance observed in this cohort persists under current standards of HIV care.

## Supporting information

S1 STROBE Statement Checklist of items that should be included in reports of cross-sectional studies. (DOC)

S2 Chart History and clinical findings, ante-mortem diagnosis of underlying cause of death compared to post-mortem diagnosis, in class I cases. (DOCX)

S1 Table Sociodemographic characteristics of HIV/AIDS patients autopsied at FMT-HVD, from 2014 to 2019. (DOCX)

S2 Table Distribution of signs and symptoms at hospital admission, length of hospital stay, comorbidities and treatment among HIV/AIDS patients autopsied at FMT-HVD between 2014 and 2019. (DOCX)

S3 Table Viral load and CD4 frequencies of 101 patients autopsied at FMT-HVD between 2014 and 2019. (DOCX)
