# Supplementary material for: Clinicopathological diagnostic concordance among autopsied adults living with HIV in the Brazilian Amazon: A retrospective cross-sectional study
Source: PLoS One. 2026 Oct 26;21(10):e0361097. doi: 10.1371/journal.pone.0361097 (PMC13704871; doi:10.1371/journal.pone.0361097)
Supplement: Supplementary file 1 [file pone.0361097.s002.docx]

**S2 Chart.** History and clinical findings, ante-mortem diagnosis of underlying cause of death compared to post-mortem diagnosis, in class I cases.

| **N°** | **Age/**  **Sex** | **Clinical history,**  **laboratory or imaging findings** | **Ante-mortem diagnosis of the underlying cause of death** | **Post-mortem diagnosis of the underlying cause of death** |
| --- | --- | --- | --- | --- |
| 1 | 28/M | HIV diagnosis 5 years ago; regular ART; symptoms of watery diarrhea and ascending paresis on admission; positive AFB; positive culture for mycobacteria; days of hospitalization: 8 | Pulmonary Tuberculosis | Disseminated cryptococcosis |
| 2 | 33/F | HIV diagnosed 5 years ago; regular ART; previously treated tuberculosis and pneumocystosis; symptoms of fever, dyspnea, vomiting and chest pain upon admission; Distended abdomen, painful on palpation and with a palpable mass on the left flank; days of hospitalization: 10 | Malignant neoplasm, without location specification | Diffuse large B-cell non-Hodgkin lymphoma |
| 3 | 25/M | Regular ART; symptoms of fever, dyspnea, cough and weight loss; days of hospitalization: 23 | Miliary tuberculosis | Kaposi’s sarcoma |
| 4 | 48/M | Previously treated tuberculosis; regular ART; symptoms of weight loss, diarrhea, oral moniliasis and asthenia; days of hospitalization: 5 | Unspecified pneumonia | Disseminated tuberculosis |
| 5 | 34/M | No ART; symptoms of fever, headache and oral moniliasis; X-rays: diffuse opacities; days of hospitalization: 8 | Pulmonary tuberculosis (no mention of bacteriological or histological confirmation) | Bacterial pneumonia |
| 6 | 29/F | HIV diagnosed 5 years ago; No ART; symptoms of dyspnea, cough and chest pain; days of hospitalization: 20 | Pneumonia | Pneumocystosis |
| 7 | 27/M | HIV diagnosed 3 months ago; regular ART; previous hospitalization with a diagnosis of treated pneumocystosis and neurotoxoplasmosis; symptoms of fever, dyspnea, chest pain and loss of strength in the lower limbs; AFB negative; days of hospitalization: 52 | Neurotoxoplasmosis | Disseminated histoplasmosis |
| 8 | 52/M | Recent HIV (admitted on the day of diagnosis); history of hypertension, DM and AMI; symptoms of hyperreflexia and left hemiparesis; AFB negative; blood culture: Enterobacter sp; days of hospitalization: 36 | Human immunodeficiency virus (HIV) disease, unspecified | Bacterial pneumonia |
| 9 | 43/M | HIV diagnosed 8 years ago, irregular ART, symptoms of fever, dyspnea, cough, weight loss, diarrhea, asthenia, low back pain radiating to the abdomen, hemoptysis; diagnostic hypotheses: Pneumocystosis, Histoplasmosis, Tuberculosis; days of hospitalization: 5 | Human immunodeficiency virus (HIV) disease, unspecified | *Strongyloides stercoralis* disseminated in lungs |
| 10 | 42/F | Irregular ART; symptoms of fever, dyspnea, asthenia; days of hospitalization: 34 | Human immunodeficiency virus (HIV) disease, unspecified | Cytomegalovirus pneumonia |
| 11 | 30/M | HIV diagnosis 4 years ago; regular ART; History of Neurotoxoplasmosis and pleural Tuberculosis; Symptoms of dyspnea, diarrhea, asthenia, pain and functional limitation in MSE; days of hospitalization: 1 | Human immunodeficiency virus (HIV) disease, unspecified | Pneumonia due to other gram-negative aerobic bacteria |
| 12 | 31/M | Recent HIV (less than 2 months ago); No ART; symptoms of coughing, vomiting, moniliasis, purulent anal discharge; submandibular lymph node enlargement; history of alcoholism, smoking, illicit substances; days of hospitalization: 1 | Respiratory tuberculosis | Neurotoxoplasmosis |
| 13 | 34/M | Recent HIV (admitted on the day of diagnosis); No ART; symptoms of fever, dyspnea, moniliasis, bacterial pneumonia; X-rays: reticular infiltrate; days of hospitalization: 1 | Pneumocystosis | Disseminated histoplasmosis |
| 14 | 30/M | Recent HIV (admitted on the day of diagnosis); No ART; symptoms of dyspnea; previous hospitalization for Tuberculosis for which he received treatment, acute kidney failure, medical hepatitis; positive AFB; days of hospitalization: 20 | Miliary tuberculosis | Cytomegalovirus pneumonia |
| 15 | 50/M | Recent HIV (admitted on the day of diagnosis); No ART; symptoms of headache, weight loss, moniliasis, alcoholism, smoking; X-rays: consolidations; AFB negative; days of hospitalization: 32 | Unspecified pneumonia | Pneumonia with foci of hemorrhage caused by Cytomegalovirus |
| 16 | 34/M | HIV diagnosed 2 months ago, regular ART use; symptoms of fever, weight loss, asthenia, myalgia; smoking history; AFB negative; days of hospitalization: 4 | Human immunodeficiency virus (HIV) disease, unspecified | Disseminated histoplasmosis |
| 17 | 36/M | Recent HIV (diagnosis one day after admission); symptoms of fever, headache, diarrhea, vomiting, asthenia, cachexia; negative blood culture; X-rays: bilateral infiltrate; days of hospitalization: 14 | Human immunodeficiency virus (HIV) disease, unspecified | Bacterial bronchopneumonia |
| 18 | 23/F | HIV diagnosed 2 years ago; regular ART; symptoms of fever, dyspnea; previous diagnosis of Tuberculosis undergoing treatment; days of hospitalization: 4 | Pulmonary Tuberculosis | Pneumocystosis |
| 19 | 45/F | HIV diagnosed 2 months ago, without ART; symptoms of fever, cough, weight loss, moniliasis, MV with bilateral snoring; days of hospitalization: 1 | Pulmonary Tuberculosis | B lymphoblastic leukemia |
| 20 | 43/F | HIV diagnosed 3 years ago; regular ART; symptoms of fever, dyspnea, cough, weight loss; previous diagnosis of malignancy (rectal cancer); diagnostic hypothesis of CMV pneumonia; days of hospitalization: 4 | Other lung disorders | Pneumocystosis |
| 21 | 32/F | Recent HIV (admitted on the day of diagnosis); symptoms of fever, cough, weight loss, diarrhea, chest pain, myalgia; days of hospitalization: 28 | Human immunodeficiency virus (HIV) disease, unspecified | Disseminated histoplasmosis |
| 22 | 38/M | HIV diagnosed 3 days ago; fever symptom; previous hospitalization for pneumonia; days of hospitalization: 12 | Unspecified bronchopneumonia | Pneumocystosis |
| 23 | 38/M | Recent HIV (admitted on the day of diagnosis); headache symptoms, moniliasis disorientation, loss of consciousness; days of hospitalization: 40 | Unspecified pneumonia | Viral encephalitis |
| 24 | 39/M | HIV diagnosed 6 months ago, without ART; cough symptoms, weight loss; blood culture and CSF positive for Escherichia coli; AFB negative; days of hospitalization: 1 | Human immunodeficiency virus (HIV) disease, unspecified | Pulmonary hemorrhage |
| 25 | 22/M | HIV diagnosed 5 years ago; Irregular ART; symptoms of fever, cough, diarrhea, moniliasis, hallucinations, neck pain; history of syphilis, pneumocystosis and tuberculosis under treatment; days of hospitalization: 4 | Pneumocystosis | Pulmonary and disseminated histoplasmosis |
| 26 | 39/F | Irregular ART; symptoms of fever, diarrhea, moniliasis, psychomotor agitation; Tuberculosis under treatment; days of hospitalization: 131 | Tuberculosis disseminated to the CNS | Viral encephalitis |
| 27 | 31/M | HIV diagnosed 2 years ago, irregular ART, symptoms of fever, dyspnea, cough, abdominal pain, asthenia; history of Neurocryptococcosis, CMV Retinitis; days of hospitalization: 7 | Unspecified bronchopneumonia | Disseminated tuberculosis |
| 28 | 50/M | Symptoms of fever, dyspnea, abdominal pain, chest pain; days of hospitalization: 16 | Unspecified bacterial pneumonia | Pulmonary histoplasmosis |
| 29 | 31/F | Regular ART; seizure symptoms; history of severe Neurotoxoplasmosis and depression with psychotic symptoms; days of hospitalization: 43 | Human immunodeficiency virus (HIV) disease, unspecified | Cytomegalovirus pneumonia |
| 30 | 23/M | HIV diagnosed less than 1 month ago; symptoms of fever, dyspnea, cough, vomiting, abdominal pain; AFB negative; X-rays: miliary pattern, diffuse interstitial infiltrate; days of hospitalization: 3 | Pulmonary Tuberculosis | Extensive lung involvement due to Histoplasmosis |
| 31 | 28/M | HIV diagnosed 8 months ago; symptoms of fever, cough, convulsion, loss of consciousness, skin lesions on the hands, feet and inguinal region D; history of acute kidney failure, esophageal candidiasis, neurological syndrome and tuberculosis under treatment; days of hospitalization: 8 | Pulmonary Tuberculosis | Pulmonary Kaposi's sarcoma |
| 32 | 31/M | HIV diagnosed 18 years ago; Irregular ART; symptoms of fever, dyspnea, diarrhea, involuntary shaking of the hands; history of neurotoxoplasmosis, hepatitis, malaria and tuberculosis under treatment; days of hospitalization: 20 | Pulmonary Tuberculosis | Lung involvement due to bacterial pneumonia and edema |
| 33 | 45/M | Recent HIV (admitted on the day of diagnosis); symptoms of fever, dyspnea, weight loss, diarrhea, abdominal pain, chest pain, lymphadenopathy, cutaneous and mucous jaundice; negative culture; days of hospitalization: 13 | HIV disease resulting in other bacterial infections | Extensive neoplastic involvement due to lymphomatoid granulomatosis |
| 34 | 48/M | HIV diagnosed 2 years ago, without ART; symptoms of headache, cough, weight loss, diarrhea; history of diabetes, tuberculosis with treatment abandonment; days of hospitalization: 6 | Pulmonary Tuberculosis | Necrotizing pneumonia due to mucormycosis |
| 35 | 43/M | HIV diagnosed 6 years ago; Irregular ART; symptoms of hyporexia and pain in the coccyx/sacral region and throughout the body; urinary and fecal incontinence; history of spastic paraplegia of unknown cause, Herpes Zoster, Dengue fever and cutaneous Leishmaniasis, Syphilis and acute kidney failure; days of hospitalization: 11 | Human immunodeficiency virus (HIV) disease, unspecified | Angiocentric lymphoma/lymphomatoid granulomatosis |
| 36 | 24/M | Recent HIV (admitted on the day of diagnosis); symptoms of dyspnea, loss of consciousness; History of Syphilis, Toxoplasmosis; hospitalization days 5 | Toxoplasma meningoencephalitis (neurotoxoplasmosis) | Pulmonary hemorrhage |
| 37 | 49/M | HIV diagnosed 1 year ago; regular ART; symptoms of fever, diarrhea, vomiting, asthenia; History of treated tuberculosis, days of hospitalization: 3 | Human immunodeficiency virus (HIV) disease, unspecified | Bronchopneumonia |
| 38 | 43/F | HIV diagnosed less than 1 month ago; symptoms of dyspnea and cough; diagnostic hypothesis of pneumocystosis; days of hospitalization: 15 | Unspecified bronchopneumonia | Pulmonary Tuberculosis |
| 39 | 22/M | HIV diagnosed 9 months ago; Irregular ART; symptoms of upper limb edema, diffuse ecchymotic lesions on the body; history of treated syphilis; diagnostic hypothesis of Kaposi's Sarcoma; days of hospitalization: 3 | Human immunodeficiency virus (HIV) disease, unspecified | Polyvisceral Kaposi's sarcoma (lung, spleen and intestine) associated with disseminated intravascular coagulation and Kasabach-Merritt phenomenon |
| 40 | 36/M | HIV diagnosed 1 month ago, without ART; symptoms of fever, dyspnea, cough, weight loss, asthenia; AFB negative; days of hospitalization: 15 | Unspecified pneumonia | Pneumocystosis |
| 41 | 42/M | HIV diagnosed 3 months ago, without ART; symptoms of fever, cough, weight loss, diarrhea, disorientation; AFB negative; X-rays: images suggestive of tuberculosis; days of hospitalization: 2 | Pulmonary Tuberculosis | Histoplasmosis in bone marrow, liver, spleen, small intestine and lung |
| 42 | 32/M | Recent HIV (admitted on the day of diagnosis); without ART, symptoms of fever, dyspnea; history of CMV, acute kidney failure, pancytopenia. days of hospitalization:7 | Unspecified pneumonia | Pneumocystosis |
| 43 | 40/M | HIV diagnosed 4 months ago; regular ART; cough symptoms, anasarcado: history of tuberculosis being treated; days of hospitalization: 7 | Pulmonary Tuberculosis | Disseminated cryptococcosis |
| 44 | 48/F | HIV diagnosed 4 days ago; symptoms of fever, cough, weight loss, diarrhea, ulcerated lesion in the MID; positive AFB; days of hospitalization: 7 | Pulmonary Tuberculosis | Pneumocystosis |
| 45 | 41/F | HIV diagnosed 5 days ago; symptoms of fever, dyspnea, cough, chest pain, severe anemia; acute kidney failure history; hospitalization days 3 | Unspecified pneumonia | Extensive lung involvement due to pneumocystosis, hemorrhage and areas of fibrosis |
| 46 | 58/M | HIV diagnosed 18 years ago; Irregular ART; symptoms of dyspnea, headache, cough; history of tuberculosis being treated; negative culture; skull tomography: extradural expansive process in C2-C3; days of hospitalization: 60 | Malignant neoplasm of the brain | Extensive pulmonary hemorrhage and edema |
| 47 | 27/M | HIV diagnosed 11 months ago; regular ART; symptoms of fever, dyspnea, headache, chest pain, palpable lymph nodes in the cervical region, edema and pain on local palpation; AFB negative; days of hospitalization: 28 | Unspecified malignant neoplasm | Extensive lung metastasis, with secondary atelectasis, due to anaplastic thyroid carcinoma |
| 48 | 29/M | Recent HIV (admitted on the day of diagnosis); symptoms of fever, dyspnea, headache, chest pain; AFB negative; X-rays: diffuse pneumonic infiltrate; days of hospitalization: 5 | Unspecified bronchopneumonia | Diffuse alveolar damage, in the organization phase, in association with an infectious process caused by pneumocystosis |
