# Supplementary material for: Clinicopathological diagnostic concordance among autopsied adults living with HIV in the Brazilian Amazon: A retrospective cross-sectional study
Source: PLoS One. 2026 Oct 26;21(10):e0361097. doi: 10.1371/journal.pone.0361097 (PMC13704871; doi:10.1371/journal.pone.0361097)
Supplement: Supplementary file 2 [file pone.0361097.s003.docx]

**Table S1:** Sociodemographic characteristics of HIV/AIDS patients autopsied at FMT-HVD, from 2014 to 2019.

| **Variables** | **N°** | **(%)** |
| --- | --- | --- |
| **Sex** | | |
| Male | 71 | 70,3 |
| Female | 30 | 29,7 |
| **Age range** | | |
| 18 to 29 | 25 | 24,7 |
| 30 to 39 | 41 | 40,5 |
| 40 to 49 | 26 | 25,7 |
| > 50 years old | 9 | 8,9 |
| **Area of residence** | | |
| North  East | 29  19 | 28,8  18,9 |
| South | 10 | 9,9 |
| South-Central | 6 | 5,9 |
| West | 5 | 4,9 |
| Midwest | 2 | 1,9 |
| Rural area | 2 | 1,9 |
| Other municipalities | 14 | 13,9 |
| N/I | 14 | 13,9 |
| **Total** | **101** | **100** |
|  | | |
| N/I: No information | | |
