# Supplementary material for: Clinicopathological diagnostic concordance among autopsied adults living with HIV in the Brazilian Amazon: A retrospective cross-sectional study
Source: PLoS One. 2026 Oct 26;21(10):e0361097. doi: 10.1371/journal.pone.0361097 (PMC13704871; doi:10.1371/journal.pone.0361097)
Supplement: Supplementary file 3 [file pone.0361097.s004.docx]

**Table S2:** Distribution of signs and symptoms at hospital admission, length of hospital stay, comorbidities and treatment among HIV/AIDS patients autopsied at FMT-HVD between 2014 and 2019.

| **Variables** | **N°** | **(%)** |
| --- | --- | --- |
| **Signs and symptoms*** |  |  |
| Fever | 49 | 48,5 |
| Dyspnea | 46 | 45,5 |
| Cough | 37 | 36,6 |
| Weight loss | 35 | 34,6 |
| Diarrhea | 29 | 28,7 |
| Vomiting | 26 | 25,8 |
| Asthenia | 25 | 24,8 |
| Chest pain | 17 | 16,8 |
| Headache | 15 | 14,8 |
| Oral moniliasis | 15 | 14,8 |
| Skin lesions | 11 | 10,8 |
| Abdominal pain | 10 | 9,9 |
| Paresis | 7 | 6,9 |
| Mental confusion | 6 | 5,9 |
| Lymph node enlargement | 5 | 4,9 |
| Seizures | 5 | 4,9 |
| Cachexia | 4 | 3,9 |
| Loss of consciousness | 4 | 3,9 |
| **Comorbidities** |  |  |
| Yes | 70 | 69,4 |
| No | 17 | 16,8 |
| N/I | 14 | 13,8 |
| **ART** |  |  |
| Regular | 40 | 39,7 |
| Irregular | 31 | 30,7 |
| No ART | 27 | 26,7 |
| N/I | 3 | 2,9 |
| **Length of hospital stay (days)** |  |  |
| 1 to 5 | 31 | 30,7 |
| 6 to 12 | 23 | 22,8 |
| 13 to 26 | 19 | 18,8 |
| 27 to 40 | 14 | 13,9 |
| 41 to 69 | 8 | 7,9 |
| > 70 to 180 | 6 | 5,9 |
| **Total** | **101** | **100** |
| N/I: No information  * subject to variation as individual patients may present with multiple clinical signs and symptoms. |  |  |
|  |  |  |
