# Supplementary material for: Clinicopathological diagnostic concordance among autopsied adults living with HIV in the Brazilian Amazon: A retrospective cross-sectional study
Source: PLoS One. 2026 Oct 26;21(10):e0361097. doi: 10.1371/journal.pone.0361097 (PMC13704871; doi:10.1371/journal.pone.0361097)
Supplement: Supplementary file 4 [file pone.0361097.s005.docx]

**Table S3:** Viral load and CD4 frequencies of 101 patients autopsied at FMT-HVD between 2014 and 2019.

| **Variable** | **N°** | **%** |
| --- | --- | --- |
| **CD4 (cells/µl)** | **86** | **85,1** |
| 2 to 200 | 73 | 72,3 |
| 201 to 500 | 12 | 11,9 |
| >500 | 1 | 0,9 |
| N/I | 15 | 14,9 |
| **Viral Load (copies/ml)** | **64** | **63,4** |
| 40 to 1.000 | 7 | 6,9 |
| 1.001 to 10.000 | 9 | 8,9 |
| 10.001 to 100.000 | 14 | 13,9 |
| 100.001 to 10.000.000 | 34 | 33,7 |
| Undetectable | 9 | 8,9 |
| N/I | 28 | 27,7 |
| **Total** | **101** | **100** |

N/I: No information
